# Supplementary material for: Intrinsic excitability mechanisms of neuronal ensemble formation
Source: eLife. 2022 May 4;11:e77470. doi: 10.7554/eLife.77470 (PMC9197391; doi:10.7554/eLife.77470)
Supplement: Transparent reporting form [file elife-77470-transrepform1.docx]

***eLife’s* transparent reporting form**

We encourage authors to provide detailed information *within their submission* to facilitate the interpretation and replication of experiments. Authors can upload supporting documentation to indicate the use of appropriate reporting guidelines for health-related research (see [EQUATOR Network](http://www.equator-network.org/%20)), life science research (see the [BioSharing Information Resource](https://biosharing.org/)), or the [ARRIVE guidelines](http://www.plosbiology.org/article/info:doi/10.1371/journal.pbio.1000412) for reporting work involving animal research. Where applicable, authors should refer to any relevant reporting standards documents in this form.

If you have any questions, please consult our Journal Policies and/or contact us: [editorial@elifesciences.org](mailto:editorial@elifesciences.org).

**Sample-size estimation**

- You should state whether an appropriate sample size was computed when the study was being designed
- You should state the statistical method of sample size computation and any required assumptions
- If no explicit power analysis was used, you should describe how you decided what sample (replicate) size (number) to use

Please outline where this information can be found within the submission (e.g., sections or figure legends), or explain why this information doesn’t apply to your submission:

Replicates samples were decided from experience and practical considerations. Number of samples and statistical tests are given in Results section and in every Figure legend, more detailed in Source Data Files associated with Figures: 1, 2, 3, 4, 5, 6, and 7.

1) Samples size were bigger than 7 neurons for every experimental group and statistical analysis. 5-7 independent biological replicated have been reported per experiment.

Outliers were not considered in the analysis but they were plotted individually using '+' marker symbol.

2) Wilcoxon-Mann-Whitney test were performed according to the sample size and statistical comparation.

**Replicates**

- You should report how often each experiment was performed
- You should include a definition of biological versus technical replication
- The data obtained should be provided and sufficient information should be provided to indicate the number of independent biological and/or technical replicates
- If you encountered any outliers, you should describe how these were handled
- Criteria for exclusion/inclusion of data should be clearly stated
- High-throughput sequence data should be uploaded before submission, with a private link for reviewers provided (these are available from both GEO and ArrayExpress)

Please outline where this information can be found within the submission (e.g., sections or figure legends), or explain why this information doesn’t apply to your submission:

The number of biological replicates is shown in Figure legends and in Source Data Files associated with Figures: 1, 2, 3, 4, 5, 6, and 7.

**Statistical reporting**

- Statistical analysis methods should be described and justified
- Raw data should be presented in figures whenever informative to do so (typically when N per group is less than 10)
- For each experiment, you should identify the statistical tests used, exact values of N, definitions of center, methods of multiple test correction, and dispersion and precision measures (e.g., mean, median, SD, SEM, confidence intervals; and, for the major substantive results, a measure of effect size (e.g., Pearson's r, Cohen's d)
- Report exact p-values wherever possible alongside the summary statistics and 95% confidence intervals. These should be reported for all key questions and not only when the p-value is less than 0.05.

Please outline where this information can be found within the submission (e.g., sections or figure legends), or explain why this information doesn’t apply to your submission:

Data analysis information is largely described in Methods section. Specificities of every data group and statistics information is detailed in Results section, Figure legends, and in Source Data Files associated with Figures: 1, 2, 3, 4, 5, 6, and 7.

Box and whisker plots (with median and 25th and 75th percentiles). Individual data points are superimposed when the replicate numbers were small and data from different groups with similar treatment were pooling.

(For large datasets, or papers with a very large number of statistical tests, you may upload a single table file with tests, Ns, etc., with reference to sections in the manuscript.)

**Group allocation**

- Indicate how samples were allocated into experimental groups (in the case of clinical studies, please specify allocation to treatment method); if randomization was used, please also state if restricted randomization was applied
- Indicate if masking was used during group allocation, data collection and/or data analysis

Please outline where this information can be found within the submission (e.g., sections or figure legends), or explain why this information doesn’t apply to your submission:

Allocation of samples in experimental groups is described in Methods section. Specificities of every data group is detailed in Results section, Figure legends and Source Data Files associated with Figures: 1, 2, 3, 4, 5, 6, and 7.

**Additional data files (“source data”)**

- We encourage you to upload relevant additional data files, such as numerical data that are represented as a graph in a figure, or as a summary table
- Where provided, these should be in the most useful format, and they can be uploaded as “Source data” files linked to a main figure or table
- Include model definition files including the full list of parameters used
- Include code used for data analysis (e.g., R, MatLab)
- Avoid stating that data files are “available upon request”

Please indicate the figures or tables for which source data files have been provided:

Relevant numerical data presented as graphs in the Figures: 1, 2, 3, 4, 5, 6, and 7, have been provided as Source Data Files.
